# A Simplified Ultrasound Comet Tail Grading Scoring to Assess Pulmonary Congestion in Patients with Heart Failure

**DOI:** 10.1155/2018/8474839

**Published:** 2018-01-02

**Authors:** Hong Li, Yi-Dan Li, Wei-Wei Zhu, Ling-Yun Kong, Xiao-Guang Ye, Qi-Zhe Cai, Lan-Lan Sun, Xiu-Zhang Lu

**Affiliations:** ^1^Department of Ultrasound, Shanxi Provincial People's Hospital, Taiyuan 030012, China; ^2^Department of Echocardiography, Beijing Chaoyang Hospital, Capital Medical University, Beijing 100020, China

## Abstract

Ultrasound lung comets (ULCs) are a nonionizing bedside approach to assess extravascular lung water. We evaluated a protocol for grading ULC score to estimate pulmonary congestion in heart failure patients and investigated clinical and echocardiographic correlates of the ULC score. Ninety-three patients with congestive heart failure, admitted to the emergency department, underwent pulmonary ultrasound and echocardiography. A ULC score was obtained by summing the ULC scores of 7 zones of anterolateral chest scans. The results of ULC score were compared with echocardiographic results, the New York Heart Association (NYHA) functional classification, radiologic score, and N-terminal pro-b-type natriuretic peptide (NT-proBNP). Positive linear correlations were found between the 7-zone ULC score and the following:* E*/*e*′, systolic pulmonary artery pressure, severity of mitral regurgitation, left ventricular global longitudinal strain, NYHA functional classification, radiologic score, and NT-proBNP. However, there was no significant correlation between ULC score and left ventricular ejection fraction, left ventricle diameter, left ventricular volume, or left atrial volume. A multivariate analysis identified the* E*/*e*′, systolic pulmonary artery pressure, and radiologic score as the only independent variables associated with ULC score increase. The simplified 7-zone ULC score is a rapid and noninvasive method to assess lung congestion. Diastolic rather than systolic performance may be the most important determinant of the degree of lung congestion in patients with heart failure.

## 1. Introduction

In patients with congestive heart failure or intravascular volume overload, redistribution of fluids within the lungs leads to pulmonary edema. Excessive extravascular lung water (EVLW) accumulates in the interstitial and alveolar spaces, due to elevated left ventricular (LV) filling pressures [1]. The most accurate methods for assessing EVLW are expensive, invasive, and impractical for routine clinical practice [2]. Thus, EVLW is often assessed by chest X-ray, although the sensitivity is low [3] and includes the risk of radiation exposure.

Lung ultrasound is a noninvasive and nonionizing imaging technique that has been previously proposed as a bedside tool for evaluating pulmonary congestion in patients with heart failure [4]. Lichtenstein and Mezière [5] reported that multiple anterior diffuse B-lines with lung sliding indicated pulmonary edema with 97% sensitivity and 95% specificity. However, a standard method to quantify the volume of EVLW by lung ultrasound has not been established, since there is no defined method for grading the severity of signs that are typical on lung ultrasound. The 28-sector approach has been applied to partially quantify lung congestion and showed positive linear correlation with the radiologic lung water score [6–9]. Yet, this comprehensive scoring method is inconvenient in daily clinical practice, and counting B-lines (also known as ultrasound lung comets or ULCs) in heart failure patients with lung congestion is confusing because B-lines often merge. Therefore, a simpler method is needed.

Echocardiography is a noninvasive tool which provides a good understanding of the functional and hemodynamic information in heart failure patients. The ability to evaluate ULCs on lung ultrasound may provide further insight into the clinical and pathophysiological involvement of the lungs. The present study assessed the performance of a simplified ULC scoring system for evaluating pulmonary congestion and investigated clinical and echocardiographic correlates of the ULC score.

## 2. Methods

### 2.1. Study Protocol

This was a prospective study. The Ethics Committee of Beijing Chaoyang Hospital approved the protocol, and all patients provided informed consent.

During a 6-month period, from August 2015 to January 2016, we studied patients with congestive heart failure who satisfied the modified Framingham criteria [10] and were admitted to our emergency department. All the patients presented with shortness of breath and were suspected of New York Heart Association (NYHA) classification II, III, or IV [11].

**Figure 1:**
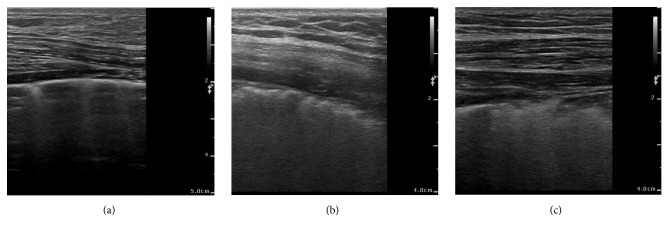
A linear probe was used to exclude noncardiac ULCs. (a) Normal pleura line and cardiac ULCs. (b, c) The abnormal pleural line could also generate ULCs (which are best visible under real-time examination), and they were confirmed by high-resolution computed tomography as interstitial lung disease and pneumonic infiltrate, respectively.

Patients with the following were excluded: age < 18 years, atrial fibrillation, mitral stenosis, and pulmonary disease. A diagnosis of pulmonary disease was based mainly on clinical symptoms, chest radiograph, and blood tests. Also excluded were patients with an abnormal pleural line observed by linear probe because B-lines can arise due to interstitial lung disease [12] or pneumonia [13] (Figure 1).

Cardiac and lung echographic examinations were performed before intravenous diuretic therapy. All patients were analyzed in the supine, near-to-supine, or lateral position. An experienced operator (HL) with 8 years of echographic examination experience and 2 years of lung ultrasound experience performed the examinations, using Philips CX50 (Philips Ultrasound, Bothell, Washington, USA) with S5-1 phased-array probe (1–5 MHz) and L12-3 linear probe (3–12 MHz).

Before the echographic examinations, a bedside anteroposterior chest X-ray was performed with the patient in the supine position. Scoring of pulmonary congestion was determined through a previously validated radiologic score incorporating assessment of variables (Table 1) [14]. The film was read by an experienced radiologist blinded to the ultrasound and clinical findings. The intra- and interobserver reproducibility of the radiologic scoring among experienced observers were very high, as previously described. In addition, a sample of blood (5 ml) was collected for blinded measurement of NT-proBNP.

### 2.2. Transthoracic Echocardiographic Study

All patients underwent transthoracic echocardiography examination at bedside. In accordance with the recommendations of the American Society of Echocardiography, the LV end-diastolic and end-systolic diameters (LVEDD and LVESD, resp.) were measured from the M-mode trace, obtained via a parasternal long-axis view. Left ventricular end-diastolic and end-systolic volumes (LVEDV and LVESV), ejection fraction (LVEF), and left atrial volume were obtained from 2-chamber and 4-chamber views using the biplane Simpson's method and indexed to body surface area. The peak Doppler velocities of early (*E*) and late (*A*) diastolic flow and the ratio of* E* to* A* (*E*/*A*) were measured from the apical 4-chamber view. A 1.5 mm sample volume was placed at the septal and lateral corner of the mitral annulus. An analysis was also performed for early (*e*′) and late diastolic velocity, and the average* E*/*e*′ ratio was calculated. The severity of mitral regurgitation was assessed semiquantitatively (i.e., mild, moderate, or severe) by color flow Doppler [15]. The systolic pulmonary artery pressure (SPAP) was calculated as the sum of the maximum systolic tricuspid regurgitation pressure gradient and the right atrial pressure. The right atrial pressure was estimated on the basis of the diameter and inspiratory collapse index of the inferior vena cava [16]. Tricuspid annular plane systolic excursion (TAPSE) was measured by M-mode echocardiography in the apical 4-chamber view as the longitudinal systolic excursion of the tricuspid annulus [17]. Right ventricular dysfunction was defined as TAPSE < 17 mm [17]. LV global longitudinal strain (GLS) analysis was performed offline using commercially available software (QLAB version 10.3; Philips Ultrasound, Seattle, USA), averaging the peak longitudinal strain of the 3 apical views. GLS data are expressed as absolute values.

### 2.3. Transthoracic Lung Ultrasound

After the transthoracic echocardiography examination, all patients underwent transthoracic lung ultrasonography with the same phased-array transducer. Seven zones were considered in our simplified ULC scoring method. The anterior chest wall was delineated from the sternum to the anterior axillary line and was subdivided into upper and lower halves, from the clavicle to the diaphragm. The lateral zone was delineated from the anterior to the posterior axillary line and was subdivided into upper and lower halves (the area above the fourth intercostal space was defined as the upper area). We initially adopted an 8-zone protocol, but inclusion of the anterior lower area on the left side was subsequently removed, because most of the study population had an enlarged heart which intervened with the area. Therefore, the 7-zone protocol was adopted.

The elementary findings that were evaluated were the ULCs (also known as B-lines), defined as hyperechogenic, vertical comet tail artifacts with a narrow base, spreading from the pleural line to the further border of the screen [9].

**Figure 2:**
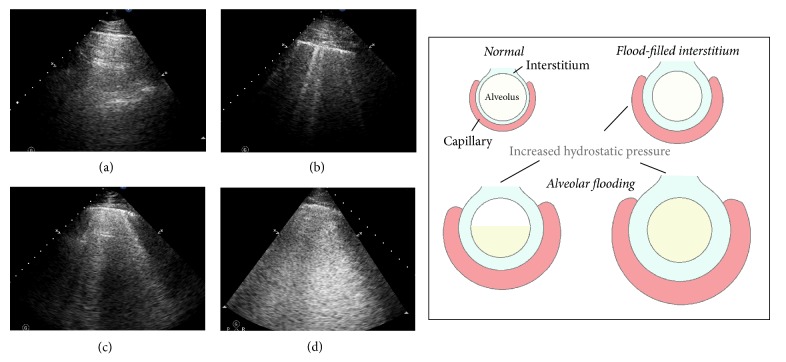
Increasing severity of interstitial or alveoli involvement. (a) Normal lung; B-lines are absent. (b) Septal syndrome; B-lines are about 7 mm apart, corresponding to subpleural septa. (c) Interstitial-alveolar syndrome; B-lines are confluent. (d) White lung. B-lines have coalesced, resulting in an echographic lung field that is almost completely white.

According to the increasing order of severity of interstitial or alveoli involvement, images were classified as zero, septal syndrome, interstitial-alveolar syndrome, or white lung [18] (Figure 2). Zero was defined as the absence of B-lines. Septal syndrome was defined as B-lines at regular distances, corresponding to pleural projection of the subpleural septa (equal to about 7 mm). In interstitial-alveolar syndrome, B-lines become more confluent, separated by <7 mm. White lung was designated for B-lines that coalesced, resulting in an almost completely white echographic lung field (confluent B-lines > 80%; Figure 2) (Videos 1–4 in Supplementary Materials). A simplified 7-zone ULC score was then calculated according to the grades: 0 = zero, 1 = septal syndrome, 2 = interstitial-alveolar syndrome, and 3 = white lung. Each intercostal space was examined thoroughly, and the images recorded in each zone were those with the highest score. All clips of transthoracic lung ultrasonography were recorded and reviewed blinded to echocardiographic data.

The stored images of each patient were scored one month after the baseline assessment, by the same investigator (HL) who performed the corresponding transthoracic ultrasonography examination. Interobserver reliability was assessed by another observer (WZ) by dynamic clips in a set of 20 cases. Each investigator was blinded to the previous results.

We also scanned the pleural line with a high-resolution linear probe, to exclude pneumogenic ULCs (Videos 5–7 in Supplementary Materials).

### 2.4. Statistical Analysis

Standard descriptive results are expressed as mean and standard deviation, and categorical data are expressed as percentage. Correlations between variables were assessed by Spearman's 2-tailed method. Independent correlates of ULC score were identified by multiple linear regression analyses after logarithmic transformation of NT-proBNP. The coefficient of differences among 3 groups was compared using one-way analysis of variance. To assess intraobserver and interobserver reliability, the ULC scores were calculated by a weighted kappa statistic. The diagnostic utility of transthoracic echocardiography in detecting moderate or severe pulmonary congestion symptoms was determined using receiver-operating characteristic (ROC) curves. The best threshold was obtained by selecting the point on the ROC curve that maximized both sensitivity and specificity. Comparisons of the values of two groups were performed using the independent-samples Student's *t*-test and Mann–Whitney nonparametric test. A *P* value < 0.05 was considered statistically significant. The statistical analyses were performed using SPSS software (version 20.0; IBM, Chicago, IL, USA) and GraphPad 5.0.

## 3. Results

During the study period, 93 heart failure patients with dyspnea were enrolled (Table 2).

### 3.1. Transthoracic Lung Ultrasound

Assessments of ULCs were performed in all patients (feasibility = 100%). Bilateral diffuse B-lines were identified in all patients by lung ultrasound [5]. The duration of the 7-zone lung ultrasound examination was 2.7 ± 0.5 minutes. The median ULC score was 9 (range: 2–20).

The kappa values for the intra- and interobserver reliabilities of the simplified ULC score assessment were 0.92 and 0.90, respectively.

### 3.2. Comparisons between NYHA Functional Class, Radiologic Score, NT-proBNP, and ULC Score

Significant linear correlations were found between the simplified ULC score and radiologic score (*r* = 0.60; *P* < 0.0001), NT-proBNP values (*r* = 0.50; *P* < 0.0001), and NYHA functional class (*r* = 0.44, *P* < 0.0001).

### 3.3. Comparison between Echocardiographic Parameters and ULC Score

The mean LVEF was 36% (range: 20%–55%; Table 2). Among the 93 patients, 8 (9%), 44 (47%), and 41 (44%) conformed to diastolic dysfunction grades I, II, and III, respectively [19]. Right ventricular dysfunction was found in 39 (42%) patients. Mitral regurgitation was recognized as mild in 46 (50%), moderate in 33 (35%), and severe in 14 (15%) patients. Tricuspid regurgitation was present in 81 patients. GLS analysis was feasible in 87 of the 93 patients (94%) who had optimal image quality for analysis. The echocardiographic characteristics of patients are reported in Table 3.

**Figure 3:**
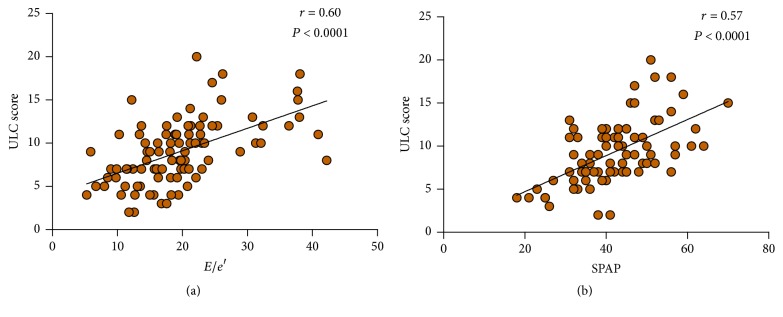
Correlation between ULC score and* E*/*e*′ (a) and systolic pulmonary artery pressure (SPAP) (b).

There was a significant correlation between the simplified ULC score and each of the following: average* E*/*e*′ ratio (*r* = 0.60, *P* < 0.0001), SPAP (*r* = 0.57, *P* < 0.0001; Figures 3(a)-3(b)), and severity of mitral regurgitation (*r* = 0.47, *P* < 0.0001). The correlation between simplified ULC score and GLS was also significant (*r* = −0.29, *P* < 0.01), but rather weak. No significant correlation was found between ULC score and any of the following parameters: LVEF, end-diastolic or end-systolic diameter, volume, or volume index. There was also no significant correlation between ULC score and left atrial volume, left atrial volume index, or TAPSE.

**Figure 4:**
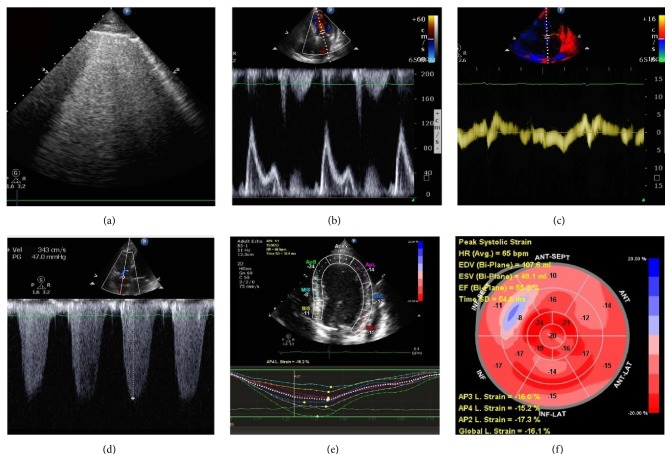
Lung ultrasound and echocardiographic parameters of a patient with congestive heart failure. (a) Interstitial-alveolar syndrome was detected by lung ultrasound. (b) Mitral inflow showed *E*/*A* > 2. (c) Tissue Doppler early (*e*′) and late (*a*′) diastolic velocities were markedly reduced. (d) Peak TR velocity by CW Doppler; peak right ventricle to right atrial systolic pressure gradient is 47 mmHg. (e, f) Global longitudinal strain analysis was −16.1%. Note that the left ventricular ejection fraction was 55.3% (f).

Figure 4 shows a patient with obvious pulmonary edema on lung ultrasound, in whom* E*/*e*′ was significantly reduced, SPAP was increased, and LV GLS was mildly reduced, but LVEF was normal.

The multivariate analysis showed that the only variables independently associated with ULC score were* E*/*e*′ (beta = 0.24, *P* < 0.01), SPAP (beta = 0.32, *P* < 0.01), and the radiologic score (beta = 0.42, *P* < 0.0001).

The patients were stratified into 3 groups by LVEF ≥ 40% (*n* = 28, 30%), 25–39% (*n* = 58, 62%), or <25% (*n* = 7, 8%). Among these groups, the ULC scores were statistically similar (*P* > 0.5). However, the ULC scores of the 3 grades of diastolic dysfunction (grades I, II, and III) were significantly different (*P* < 0.01; Table 4).

The optimal cutoff value of ULC score according to the ROC curve was 8 for predicting NYHA ≥ 3 (i.e., moderate or severe congestion symptoms according to NYHA [11]). The area under the curve was 0.85 (*P* < 0.0001), and the sensitivity and specificity was 78.6% and 76.9%, respectively. Compared with patients with ULC < 8, those with ULC ≥ 8 were older, with higher* E*/*e*′, SPAP, radiologic score, NT-proBNP, LV diastolic function, and NYHA functional class and more severe mitral regurgitation (Table 5).

**Figure 5:**
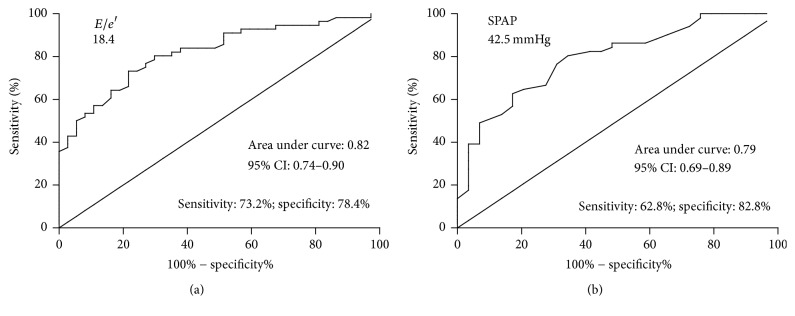
ROC curves showing the diagnostic performance of* E*/*e*′ and SPAP for predicting ULC score ≥ 8. (a) ROC curve of the* E*/*e*′ and (b) ROC curve of the SPAP. SPAP: systolic pulmonary artery pressure.

An ROC curve was plotted to predict further for moderate-to-severe pulmonary congestion symptoms. (i.e., ULC score ≥ 8; Figure 5). The optimal cutoff value for* E*/*e*′ according to the ROC curve was 18.4 (area under the curve: 0.82, *P* < 0.0001; Figure 5(a)). The sensitivity and specificity were 73.2% and 78.4%, respectively. The optimal cutoff value for SPAP based on the ROC curve was 42.5 mmHg for predicting moderate-to-severe pulmonary congestion (area under the curve: 0.79, *P* < 0.0001; Figure 5(b)), with a sensitivity and specificity of 62.8% and 82.8%, respectively.

## 4. Discussion

The primary objective of the present study was to evaluate a simplified protocol for scoring ULCs to estimate pulmonary congestion in heart failure patients. This 7-zone protocol was compared with the echocardiographic results, NYHA functional classification, radiologic score, and NT-proBNP. We found that the 7-zone ULC score correlated with LV diastolic functional parameters, SPAP, GLS, the severity of mitral regurgitation, NYHA functional classification, radiologic score, and NT-proBNP. Multivariate analysis identified the* E*/*e*′, systolic pulmonary artery pressure, and radiologic score as the only independent variables associated with ULC score increase among echocardiographic results. It is important to note that the patients enrolled in the study were patients with heart failure and varying degrees of dyspnea.

ULCs are multiple comet tails and a simple echographic sign of EVLW [9]. Normal extravascular lung water is <500 mL or <10 mL/kg [20, 21], while excessive EVLW leads to interstitial and alveolar edema, that is, lung congestion [22]. The distribution of ULCs is a reflection of the volume of pulmonary congestion [18]. When decompensated congestive heart failure occurs, increased LV end-diastolic pressure and left atrial pressure lead to elevated pulmonary venous pressure and then increased hydrostatic pressure in the pulmonary capillaries. Mild elevation of left atrial pressure (18–25 mmHg) causes edema in the interstitial spaces and thickened subpleural interlobular septa. When left atrial pressure rises further (>25 mmHg), the lymphatic resorption capacity is exceeded and edema fluid breaks through the lung epithelium and pours into the alveoli [23]. The density and distribution of ULCs will vary according to the pathologic states described above: scattered septal syndrome represents thickened subpleural interlobular septa. Interstitial-alveolar syndrome and white lung are a more severe form of interstitial lung syndrome, that is, alveolar flooding [18]. In the latter stage, gas exchange is impaired and dyspnea becomes more serious. Therefore, the correlation between ULC score and severity of dyspnea, in accordance with the NYHA functional classification, makes rational sense.

Serum NT-proBNP is a biomarker that is widely used for diagnosing heart failure, although the sensitivity and specificity are not perfect. The use of NT-proBNP coupled with lung ultrasound could significantly improve the diagnostic accuracy in determining heart failure [24]. The presence of B-profile or the number of B-lines in lung ultrasound that correlate with higher BNPs levels was previously studied [25, 26]. We used a 7-zone scanning protocol that was compared with NT-proBNP and also detected the positive correlation between them.

A recent study used a simplified 4-sector method and reported good correlation with EVLW values derived from transpulmonary thermodilution [27]. Restricting the examination to the anterior pulmonary surface is likely sufficient for critically ill patients in intensive care. However, in our opinion, applying transthoracic ultrasonography in cardiological settings requires the anterolateral pulmonary surface as well. It was also suggested by Liteplo et al. [28] that an 8-zone protocol is strongly predictive for patients with congestive heart failure, and another simplified method with an 8-zone protocol using ultrasound was reportedly predictive of cardiogenic lung edema [29]. In the present study, we adopted a 7-zone protocol because we found that most of the patients enrolled in our study had enlarged heart which interfered with the view of the anterior lower area on the left side.

For patients with suspected heart failure, echocardiography is an essential imaging tool that can be used to measure LV systolic and diastolic function, estimate pulmonary capillary wedge pressure (PCWP) and SPAP, and evaluate LV filling pressure [30]. Existing literature is sparse regarding the association between lung congestion on lung ultrasound and cardiac function and structure, and in most studies the population was not restricted, or GLS assessment of LV was not included. A previous study found a correlation between the number of B-lines and LVEF, or B-lines and the degree of diastolic dysfunction in patients with suspected heart failure [6]. In a study of 72 patients (53 and 19 with LV systolic dysfunction and normal function, resp.), Agricola et al. [31] reported a positive linear correlation between B-lines and* E*/*e*′, estimated PCWP, SPAP, and LVEF. Another report corroborated this finding, in addition to a significant linear correlation between B-lines and left atrial volume and pulmonary pressure in a cohort of dialysis patients [1]. These data are in broad agreement with the present study, in which* E*/*e*′, a surrogate marker for left-side filling pressures and SPAP due to pulmonary venous congestion, had the strongest association with ULCs.

However, unlike the above studies, we found that the LVEF was somewhat less informative for predicting the degree of lung congestion. The discrepancy in results could be due to different study populations. Ours was limited exclusively to a cohort of identified left heart failure patients with pulmonary congestion. In some contexts, it has been shown that the signs and symptoms of congestive heart failure correlate poorly with LVEF [32, 33]. Gandhi et al. [34] proposed that, in patients with acute hypertensive pulmonary edema, the edema was due to the exacerbation of diastolic dysfunction, but not to systolic dysfunction. It was also reported in a study of patients with cardiogenic pulmonary edema detected by lung ultrasound that up to 15.4% had an LVEF > 50%, and it was thought that diastolic dysfunction may be the cause of pulmonary edema [29]. Actually, as a parameter of LV systolic function, LVEF is load-dependent, linked to the quality of imaging and LV geometry, and may not correlate with functional status. Interestingly, although GLS weakly correlated with ULC score in the present study, the multivariate analysis showed no independent association. Therefore, this finding further validates that systolic performance may not be better than the diastolic one for determining the degree of lung congestion in patients with heart failure.

An enlarged left heart is suggestive of chronically elevated LV filling pressure. A normal left heart volume is often noted in patients with acute increase in LV filling pressures or in the earliest stage of diastolic dysfunction. However, our study population included patients with a history of chronic heart failure; and this may be the reason for the inconsistency between left heart volume and pulmonary congestion degree.

In the present study, we offer a simplified ULC scoring method to estimate the degree of congestion that considered only 7 thoracic zones. This tool is less refined than counting all the B-lines in 28 sectors but provides easy-to-acquire data in an emergency setting, and it is much easier to differentiate 4 types of ULC patterns than it is to count B-lines.

The study may be considered limited by the lack of patients with atrial fibrillation and mitral stenosis. A goodly proportion of patients with heart failure experience these conditions, especially those with preserved LVEF [35]. On the other hand, our relatively homogeneous population avoided the possibility of outpatients with poor echocardiographic indices or patients receiving intravenous diuretics.

Our conclusions are based only on imaging evaluations and echocardiographic indices. While B-lines are thought to reflect EVLW, there is no reference standard available to verify the EVLW volume and left atrial pressure (LAP) by invasive catheter.

## 5. Conclusion

This novel simplified ULC scoring method is a rapid, noninvasive, and reliable tool to assess pulmonary congestion in patients with heart failure. Diastolic rather than systolic performance may be the most important determinant of the degree of lung congestion in these patients.

## Figures and Tables

**Table 1:** Radiologic score variables.

Variables	Score		
	Mild	Moderate	Severe
Hilar vessels			
Enlarged	1	2	3
Increased in density	2	4	6
Blurred	3	6	9
Kerley lines			
A	4	8	
B	4	8	
C	4	8	
Micronodules	4	8	
Widening of interlobar fissures	4	8	12
Peribronchial and perivascular cuffs	4	8	12
Extensive perihilar haze	4	8	12
Subpleural effusion	5	10	
Diffuse increase in density	5	10	15

**Table 2:** Patients' clinical characteristics.

Variables	Mean ± SD or number (%)
Subjects, *n*	93
Age, y	67 ± 14
Gender, female/male	32/61
Body surface area, m^2^	1.8 ± 0.2
Hypercholesterolemia	40 (43)
Diabetes	20 (22)
Previous MI^a^	16 (17)
PCI^b^	10 (11)
CABG^c^	4 (4)
NT-proBNP, pg/ml	11645 ± 11070
Radiologic score	13 ± 7
NYHA functional class	
II	28 (30)
III	56 (60)
IV	9 (10)
Cause of heart failure	
Coronary artery disease	64 (69)
Hypertension	14 (15)
Dilated cardiomyopathy	7 (8)
Myocarditis	4 (4)
Perinatal cardiomyopathy	1 (1)
Autoimmunity cardiomyopathy	1 (1)
Alcoholic cardiomyopathy	2 (2)

^a^MI: myocardial infarction; ^b^PCI: percutaneous coronary intervention; ^c^CABG: coronary artery bypass grafting.

**Table 3:** Patients' echocardiographic characteristics.

Variables	Mean ± SD
LV ejection fraction, %	35.7 ± 7.8
LV end-diastolic diameter, mm	60.1 ± 7.4
LV end-systolic diameter, mm	47.4 ± 8.6
LVEDV, mL/LVEDV_index_, mL/m^2^	166.3 ± 47.5/93.4 ± 25.5
LVESV, mL/LVESV_index_, mL/m^2^	108.3 ± 38.7/60.9 ± 21.4
LAV, mL/LAV_index_, mL/m^2^	78.1 ± 21.2/44 ± 12
SPAP, mmHg	42.2 ± 10.3
TAPSE, mm	17.7 ± 4.3
GLS, %	9.2 ± 2.7

LV: Left ventricular; LVEDV: left ventricle end-diastolic volume; LVESV: left ventricle end-systolic diameter; LAV: left atrial volume; index: divided by BSA (body surface area); SPAP: systolic pulmonary artery pressure; TAPSE: tricuspid annular plane systolic excursion; GLS: global longitudinal strain.

**Table 4:** ULC scores by diastolic function grade and left ventricle ejection fraction (LVEF).

		ULC score	*P*
LV diastolic function grade	Grade I	5.6 ± 2.4	<0.001
	Grade II	8.4 ± 3.2	
	Grade III	10.3 ± 3.7	

LV ejection fraction (LVEF)	LVEF ≥ 40%	8.5 ± 3.0	0.52
	LVEF 25–39%	9.1 ± 3.8	
	LFEF < 25%	10.1 ± 4.4	

**Table 5:** Patients with ULC scores < 8 and ≥8^*∗*^.

Variables	ULC score		*P*
	<8 (*n* = 37)	≥8 (*n* = 56)	
*E*/*e*′	14.6 ± 4.5	22.7 ± 8.1	<0.0001
SPAP, mmHg	35.2 ± 8.6	45.8 ± 9.3	<0.0001
GLS, %	9.9 ± 2.2	9.1 ± 2.7	0.14
LV ejection fraction, %	36.3 ± 7.6	35.3 ± 8.0	0.55
LVEDD, mm	59.3 ± 7.6	60.6 ± 7.3	0.46
LVEDV_index_, mL/m^2^	91.6 ± 22.8	94.6 ± 27.3	0.57
LAV_index_, mL/m^2^	43.4 ± 13.4	44.6 ± 11.3	0.66
TAPSE, mm	18.9 ± 4.6	17.0 ± 4.0	0.06
LV diastolic function grade			<0.05
Grade I	7 (19)	1 (2)	
Grade II	18 (49)	26 (46)	
Grade III	12 (32)	29 (52)	
Mitral regurgitation			<0.001
Mild	27 (73)	19 (34)	
Moderate	8 (22)	25 (45)	
Severe	2 (5)	12 (21)	
NYHA functional class			<0.001
II	20 (54)	11 (20)	
III	15 (41)	33 (59)	
IV	2 (5)	12 (21)	
Age, y	63.6 ± 13.7	70.1 ± 13.4	<0.05
NT-proBNP, pg/ml	5046.2 ± 4325.3	15426.3 ± 12140.2	<0.0001
Radiologic score	8.2 ± 4.4	15.7 ± 6.5	<0.0001

^*∗*^Data are mean ± SD or number (%). SPAP: systolic pulmonary artery pressure; GLS: global longitudinal strain; LVEDD: left ventricle end-systolic diameter; LVEDV: left ventricle end-diastolic volume; LAV: left atrial volume; index: divided by body surface area; TAPSE: tricuspid annular plane systolic excursion; NYHA: New York Heart Association.

## Supplementary Materials

Videos 1–4. Normal lung and three types of ULCs in ultrasound images which indicate increasing severity of interstitial or alveoli involvement.

Supplementary 1 Video 1: Normal lung; B-lines are absent.

Supplementary 2 Video 2: Septal syndrome; B-lines are scattered (about 7 mm apart) corresponding to the distance of subpleural septa.

Supplementary 3 Video 3: Interstitial-alveolar syndrome; B-lines are confluent.

Supplementary 4 Video 4: White lung; B-lines have coalesced, resulting in an echographic lung field that is almost completely white.

Note. Classification of images is according to the distribution of B-lines with the “highest” score in the respiratory cycle (Videos 2–4).

Videos 5–7. Linear probe was used to exclude noncardiac ULCs (abnormal pleural line could also generate ULCs).

Supplementary 5 Video 5: Normal pleura line and cardiac ULCs.

Supplementary 6 Video 6: Fringed pleural line indicates interstitial lung disease.

Supplementary 7 Video 7: Irregular pleural line with microconsolidations.
